# Transfemoral recanalization of occluded TIPS

**DOI:** 10.1186/s42155-022-00304-3

**Published:** 2022-06-22

**Authors:** Tatjana Dell, Ulrike Attenberger, Christian Jansen, Julian A. Luetkens, Michael Praktiknjo, Daniel Kütting, Carsten Meyer

**Affiliations:** University Hospital Bonn, Bonn, Germany

**Keywords:** TIPS, Occlusion, TIPS recanalization, Transfemoral

## Abstract

**Purpose:**

To report the safety and efficacy of percutaneous transfemoral venous recanalization of occluded intrahepatic portosystemic stents (TIPS) in cases where the transjugular approach is not feasible.

**Materials and Methods:**

Between 2000 and 2020, 8 patients with occluded TIPS underwent recanalization via a percutaneous transfemoral venous access. Prior recanalization via a typical transjugular approach was attempted in all cases. Primary technical success was defined as successful crossing of the occlusion. Secondary technical success was defined as the rate of successful TIPS recanalization. Periprocedural complications were evaluated to assess procedural safety.

**Results:**

In 8/8 patients transfemoral venous TIPS recanalization was successful. No procedure-related complications were observed.

**Conclusions:**

The transfemoral venous approach is a safe and efficient alternative for TIPS recanalization in cases where the transjugular approach is not feasible.

## Background

Transjugular intrahepatic portosystemic shunt (TIPS) is a cornerstone in the treatment of portal hypertension-related complications in selected patients with a 90% success rate for decompressing portal hypertension (Tripathi et al. 2004). Even though introduction of PTFE-covered stents reduced stent dysfunction rate, it may still be as high as 30% (Weber et al. 2015) prompting reintervention if shunt occlusion or stenosis develops. In these cases**,** TIPS recanalization is typically performed via a transjugular approach. In cases presenting with total TIPS occlusion**,** canulation of the hepatic venous end of the TIPS represents the technically most challenging and in some cases impossible step. Various techniques have been reported for recanalization of occluded TIPS, in cases where conventional transjugular crossing is not feasible. Alternative recanalization techniques employing the Rösch-Uchida Stiffening Cannula, the Colapinto technique, a combined transvenous transhepatic approach or invasive a transsplenic approach may be applied (Spiliopoulos et al. 2018; Miraglia et al. 2013).

**Fig. 1:**
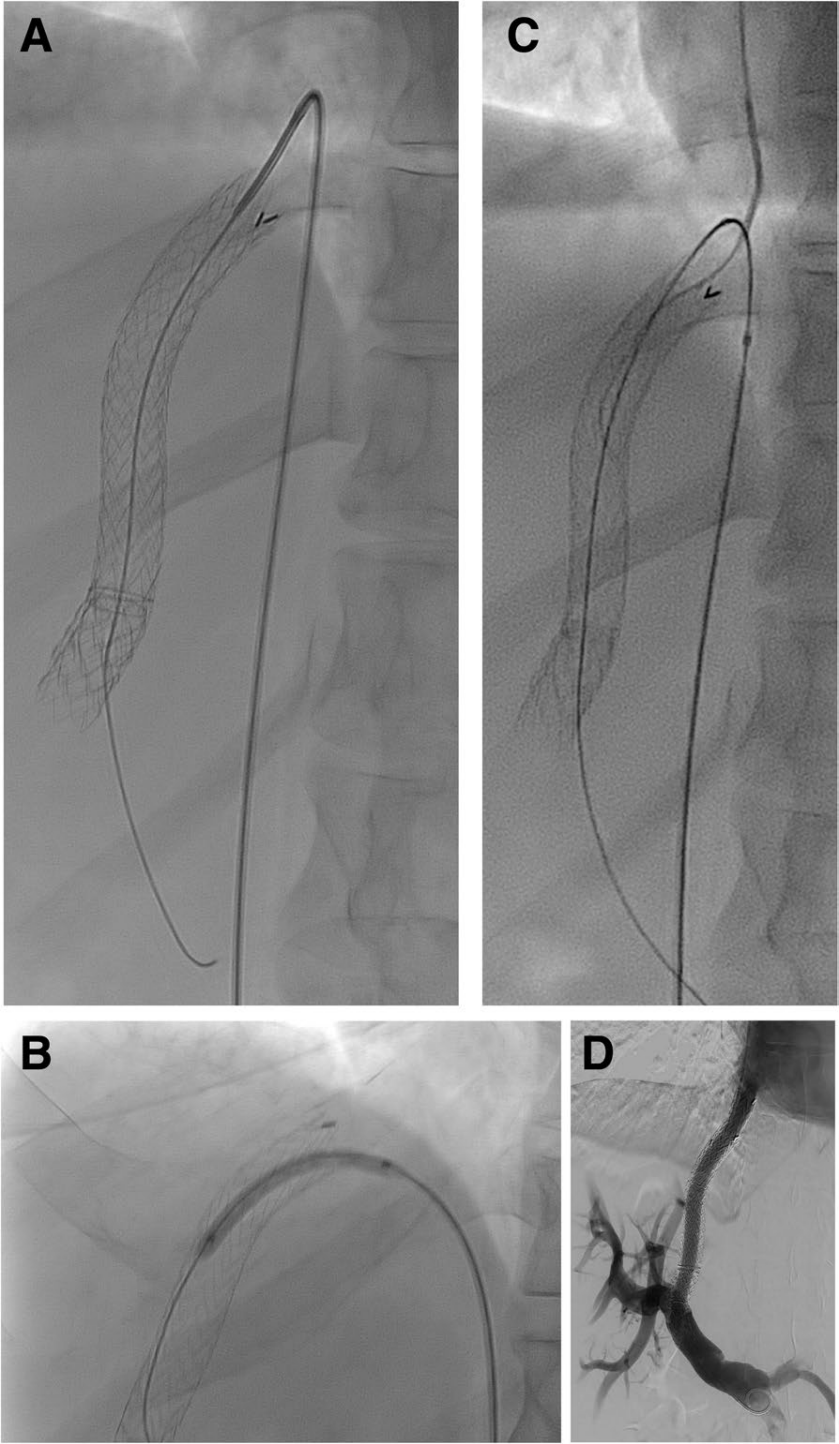
Recanalization of occluded intrahepatic portosystemic covered stent in a 41-year-old patient with Budd-Chiari syndrome where the transjugular approach was not feasible. **A** Transfemoral insertion of a reverse-curve catheter (5 Fr Simmons 1 catheter; Cordis) into the right liver-vein ostium. A hydrophilic guide wire (Terumo, Japan) was used to cross the occlusion, while maintaining tension on the catheter to ensure support while manipulating the wire through the occlusion. **B** Next, PTA was performed using a 4 mm balloon via the femoral access, which then **C** enabled transjugular insertion of a 4 Fr Vertebralis catheter into the TIPS/portal system; which had not been possible before. **D** Post-procedure portal venogram shows the restoration of the shunt function after balloon angioplasty and implantation of an additional stent-graft

We report our single-center experience in a series of challenging TIPS recanalizations via a percutaneous transfemoral venous approach allowing subsequent passage of the standard angiographic materials via a transjugular approach (Fig. 1).

## Materials and Methods

All occlusion associated TIPS revisions between January 2000 and December 2020 at our institution were retrospectively reviewed, to identify cases where recanalization was attempted via a transfemoral venous apporach. Shunt occlusions were detected during follow-up either by duplex ultrasound or by computed tomography.

Technical success was defined as successful transvenous canulation of occluded TIPS, following by successful TIPS recanalization**.** Periprocedural complications were evaluated according to the Society of Interventional Radiology (SIR) reporting standards to assess procedural safety.

All patients gave informed consent to the procedure. The retrospective study was approved by the institutional review board of the University Bonn that waived informed consent to publish and use of procedural data.

## Procedure

In all cases an initial attempt of standard transjugular recanalization of occluded TIPS had failed. The angulation of the proximal end of the stent in relation to the right atrium was unfavourable (under 90 degrees) in all cases, therefore, sufficient push could not be applied via a jugular approach to manipulate the wire through the occlusion. A right femoral venous access was established. A reverse-curve catheter (5 Fr SOS Omni; AngioDynamics, Inc, Queensbury, NY, USA (*n* = 6); 5 Fr Simmons 1 catheter; Cordis Endovascular, Miami Lakes, FL, USA (n = 2)) was advanced into the proximal IVC, turned in the direction of the right liver-vein ostium and then drawn back to canulate the proximal TIPS. Next, a hydrophilic guide wire (Radiofocus Guide Wire, Terumo Corporation, Tokyo, Japan) was used to cross the occlusion, while maintaining tension on the catheter to ensure support while manipulating the wire through the occlusion.

Percutaneous transluminal angioplasty (PTA) was performed using a 4 mm balloon (Mustang, Boston Scientific, Malborough, Mass, USA) via the femoral access. The procedure was then continued via a transjugular access by insertion of a 4 Fr Vertebralis catheter (Cordis Endovascular, Miami Lakes, FL, USA) followed by a 5 Fr Pigtail catheter (Cordis Endovascular, Miami Lakes, FL, USA) into the TIPS/portal system. The portal venous pressure was measured, and an angiogram of the portal vein with the TIPS was acquired. The portosystemic gradient (PSG) was calculated. Thereafter, an 8- or 10-mm balloon catheter (Mustang®, Boston Scientific, Marlborough, MA, USA) was used to dilate the TIPS lumen depending on the diameter of the implanted TIPS. Additional stent placement (5/8) was performed when recanalization was insufficient as visually assessed by the operator or when the PSG was still elevated (PSG > 12 mmHg). The post-interventional PSG was then subsequently calculated. In all cases, TIPS was recanalized with a satisfactory result, as demonstrated by final digital subtraction angiography. Manual compression was used for haemostasis. All patients received risk factor-dependent anticoagulation therapy.

## Results

Between January 2000 and December 2020, 81 patients underwent TIPS revisions due to complete occlusion. All patients had received a dedicated stentgraft (GORE VIATORR; Flagstaff, AZ, USA). Shunt occlusions were detected during follow-up either by duplex ultrasound or by computed tomography (CT). In 11 patients standard transjugular recanalization was not possible; in 8 of these patients transfemoral recanalization was attempted. Patient demographics and procedure details are shown in Table 1.

**Table 1:** Patient demographics and procedural details (Optional)

Variable	n (%)
Patients	8
Unterlying liver disease	
alcohol-related cirrhosis	5/8 (62,5)
cirrhosis related to hepatitis C infection	2/8 (25)
Budd-Chiari syndrome	1/8 (12,5)
Stent type	
Covered	8/8 (100)
Occlusion type	
Complete thrombosis	8/8 (100)
Time of the TIPS closure after initial stent placement	**16 months (range: 6–29)**
Indication for initial TIPS	
Variceal bleeding	3/8 (37,5)
Refractory ascites	5/8 (62,5)
Initial TIPS right jugular vein access	8/8 (100)
Technical success	8/8 (100)
Balloon angioplasty	3/8 (37,5)
Additional stent placement	5/8 (62,5)
Curved catheter type (5 Fr)	
SOS Omni Selective I catheter (AngioDynamics)	6/8 (75)
Simmons 1 catheter (Cordis)	2/8 (25)
Pre-procedural mean pressure gradient (mmHg)	15.5 ± 3.7
Post-procedural mean pressure gradient (mmHg)	7.5 ± 3.1
Complication	0/8 (0)

Primary and secondary technical success were 100%. In 3 patients PTA sufficed, 5 patients required additional stent placement (four VIATORR stents and one LIFESTREAM™ stent, BD BARD Peripheral Vascular, Tempe, Arizona) to restore TIPS flow. The portosystemic gradient could be decreased from 15.5 mmHg (range) (measured after initial 4 mm PTA) to 7.5 mmHg (range). No procedure-related complications were observed. After restoration of flow no further occlusions nor partial occlusions were noted during follow up (mean: 34 months; range: 6–122 months).

## Discussion

TIPS occlusion is commonly preceded by thrombosis in the acute setting or TIPS stenosis which in covered stents is still caused by pseudointimal hyperplasia at the edge to the hepatic parenchymal tract (Yang et al. 2010). The standard recanalization technique includes jugular vein access. After regaining access to the portal venous system**,** thrombolysis, mechanical thrombectomy, balloon dilation or self-expandable stent placement may be performed depending on the underlying pathology. Alternative techniques for therapy-refractory TIPS occlusions have been described including liver vein access using a Colapinto needle (Cook Medical, Bloomington, USA) enabling more suport for manipulation, direct stent puncture via a Rosch-Uchida needle (Cook Medical, Bloomington, USA), transhepatic stent puncture, creation of a parallel new shunt, creation of a new shunt by the ‘‘gunsight’’ approach, creation of a new shunt by direct cavoportal puncture, transhepatic direct TIPS puncture with pull-through transjugular access, and transsplenic TIPS recanalization with pull-through (Spiliopoulos et al. 2018).

Most TIPS reinterventions due to complete occlusion are accessible via the standard transjugular approach; only fourteen percent of all TIPS reinterventions performed at our institution could not be recanalized via this approach. However, especially in institutions where TIPS is frequently performed, intractable TIPS occlusions are no rarety; thus alternative methods for recanalization are necessary. The current results indicate that tranfemoral TIPS revision is a minimally invasive alternative in cases where the transjugular approach has failed. In total TIPS occlusion one of the most challenging step is canulation of the hepatic venous end of the occluded TIPS. Whether canulation is possible via a standard jugular approach depends on several factors, for example, proximal stent location and angulation in relation to the IVC (Tanaka et al. 2011). Especially when the orientation of the hepatic venous end of the TIPS is horizontal or the transition to the IVC is too sharply angled, necessary push cannot be applied via a transjugular approach. Using a reversed curved catheter with the tip fixated in the proximal stent ostium allowed for enough push to manipulate the wire through the occlusion. The percutaneous transfemoral approach was employed to enable transjugular access. The subsequent main part of the procedure was performed via **a** transjugular approach, as additional stent placement was required in the majority of patients. In our experience, transfemoral insertion of long sheaths/stents for TIPS revision is not practical. Continuing the intervention via a transjugular approach after initial recanalization is significantly easier and reduces intervention time.

Minimally invasive alternatives are sparse. Although several techniques have been reported for the catheterization of occluded TIPS, they are all relatively invasive. The transfemoral venous approach carries less traumatic risk compared to the Rosch-Uchida needle technique or other techniques employing trocar devices, where the graft material or nearby structures such as liver parenchyma, the caval wall or the atrial wall could potentially be punctured (Spiliopoulos et al. 2018); this is highlighted by the fact that unlike the transfemoral technique none of the reported alternatives were free of procedure-related complications. The Colapinto needle technique may only be employed after canulation of the proximal TIPS has been achieved, something that was not possible in any of our reported cases. Moreover, the transfemoral approach was well tolerated by all patients using only local analgesia without sedation. In comparison the transhepatic direct TIPS puncture technique as well as the transsplenic recanalization technique require general anesthesia (Habib et al. 2015).

Limitations of this study include the small sample size analyzed and including patients with favourable stent angle for transfemoral access as well as the retrospective nature, single-centre design which does not allow for the comparison among different recanalization techniques.

## Conclusion

The percutaneous transfemoral venous technique is safe and efficient. Due to its’ minimally invasive nature this technique should be taken into consideration in intractable TIPS occlusion before more invasive alternatives are employed.

## Data Availability

The datasets analysed during the current study are available from the corresponding author on reasonable request.

## Abbreviations

PTA: Percutaneous transluminal angioplasty
TIPS: Transjugular intrahepatic portosystemic shunt
SIR: Society of Interventional Radiology
PSG: Portosystemic gradient
